# Pragmatic randomised evaluation of stable thoracolumbar fracture treatment outcomes (PRESTO): study protocol for a randomised controlled feasibility trial combined with a qualitative study and survey

**DOI:** 10.1186/s40814-020-00574-5

**Published:** 2020-03-13

**Authors:** Elizabeth Cook, Alison Booth, Elizabeth Coleman, Arabella Scantlebury, Catriona McDaid, Catherine Hewitt, Belen Corbacho, Amar Rangan, Joy Adamson, Arun Ranganathan, Almas Khan, Sashin Ahuja, Emma Turner, Peter May, Catherine Hilton, David J. Torgerson

**Affiliations:** 1 York Trials Unit, Department of Health Sciences, University of York, Heslington, York, YO10 5DD UK; 2 South Tees Hospitals NHS Foundation Trust, The James Cook University Hospital, Middlesbrough TS4 3BW, North Yorkshire, UK; 3 Bart’s Health NHS Trust, The Royal London Hospital, Whitechapel Road, London, E1 1BB UK; 4 Leeds Teaching Hospitals NHS Trust, Leeds General Infirmary, Leeds, LS1 3EX West Yorkshire UK; 5 Cardiff & Vale University Health Board, University Hospital of Wales, Cardiff, CF14 4XW Wales, UK; 6 York Trials Unit and NIHR RDS YH, Department of Health Sciences, Faculty of Science, ARRC Building, University of York, Heslington, York, YO10 5DD UK

**Keywords:** Thoracolumbar, Fracture, Surgical fixation, Randomised controlled trial, Qualitative, Survey, Feasibility, Pilot

## Abstract

**Background:**

A thoracolumbar fracture is the most common fracture of the spinal column. Where the fracture is not obviously stable or unstable, the optimal management is uncertain. There are variations between surgeons, treating centres and within the evidence base as to whether surgical or non-surgical approaches should be used. In addition, the boundaries of this zone of uncertainty for stability are unclear.

This study has been designed in response to an NIHR HTA commissioning brief to assess the feasibility of undertaking a large-scale trial to evaluate the effectiveness of surgical and non-surgical treatments for thoracolumbar fractures without neurological deficit.

**Methods:**

Assessment of feasibility will be addressed through three elements: a randomised external feasibility study, a national survey of surgeons and a qualitative study.

The external feasibility study is a pragmatic, parallel-group, randomised controlled trial comparing surgical fixation (intervention) versus non-surgical management (control). Recruitment will take place in three secondary care centres in the UK.

The primary outcome is recruitment rate, defined as the proportion of eligible participants who are randomised. Further outcomes related to recruitment, randomisation, drop-out, cross-over, loss to follow-up, completeness of outcome data, study processes and details of the interventions delivered will be collected.

The survey of surgeons and qualitative study of clinicians, recruiting staff and patients will enhance the feasibility study, enabling a broad overview of current practice in the field in addition to perceived facilitators and barriers to running a full-scale trial.

**Discussion:**

PRESTO is a feasibility study which aims to inform methodology for a definitive trial comparing surgical fixation with non-surgical management for patients with stable thoracolumbar fractures.

**Trial registration:**

The trial is registered with the International Standard Randomised Controlled Trial Register (ISRCTN12094890). Date of registration was 22/02/2018 (http://www.isrctn.com/ISRCTN12094890).

## Background

Thoracolumbar fractures are the most common fracture of the spinal column [1]. The potential consequences for people who experience a thoracolumbar fracture include pain, loss of function impacting on the ability to work and undertake other activities, spinal deformity (kyphosis), and in some cases, paralysis.

There appears to be informal consensus that simple compression fractures without neurological complications can be managed without surgery [2], and this is reflected in UK practice. Similarly, obviously unstable fractures where there is neurological damage or an elevated risk of damage will require surgical fixation. However, there is a zone where there is variation between surgeons and between centres as to whether surgical or non-surgical approaches should be used. In addition, there is uncertainty in the evidence base as to the most effective treatment in terms of pain, speed of recovery, return to normal activities, prevention of kyphosis, and any associated problems with chronic back-pain and balance. The boundaries of this zone of uncertainty are unclear.

The funder commissioning brief requested inclusion of patients with high and low energy fractures. Although there are differences between the populations, in the context of a pragmatic trial, and with the implementation of stratified randomisation (high-energy trauma fractures and osteoporotic fractures) and appropriate analysis, it may be possible to include both groups in the same trial. Equally, there is likely to be differences in opinion amongst spinal surgeons regarding the acceptability of such a trial. Therefore, testing this in a feasibility study is important and we propose to include high-energy trauma and low-energy osteoporotic fractures.

A Cochrane review [3], evaluating the evidence on the effectiveness of surgical and non-surgical treatments for thoracolumbar burst fractures without neurological deficit, concluded that there is a need for a large, multicentre, high-quality and adequately reported randomised controlled trials (RCT) of such interventions to address the evidence gap for these types of fractures. This study, designed in response to a NIHR HTA commissioning brief, will assess the feasibility of undertaking such a large-scale randomised clinical trial.

## Aims and objectives

The aim of this study is to establish whether it is feasible to deliver a trial comparing surgical fixation to initial non-surgical management for patients with a stable thoracolumbar fracture without spinal cord injury.

The study will address the following specific questions:

1. Are surgeons willing to randomise eligible patients and adhere to the allocation to (i) surgical fixation or (ii) initial non-surgical management?
2. Are patients willing to be randomised and adhere to the allocation in a trial comparing the two treatments?
3. What is the completeness of follow-up in this population?
4. Are there a sufficient number of centres and surgeons (with sufficient caseloads of eligible patients) willing to participate in a future RCT to make the trial feasible within a viable timescale?
5. What methods of establishing spinal stability and suitability for surgery or non-surgical management are currently used?
6. What methods of surgical fixation and non-surgical management are currently being used?
7. What are the barriers to successful delivery of a future trial and how can they be overcome?
8. Can the British Spine Registry be used to collect participant data in a trial?
9. What is the most suitable primary endpoint for a main trial?
10. How can we accurately identify, quantify and value economic data to capture the impact of the two treatments on the NHS and productivity?

## Design

The trial objectives will be addressed through three elements:

i. a randomised external feasibility trial
ii. a national survey of orthopaedic spine surgeons
iii. a qualitative study including clinicians, recruiting staff and patients

## Methods: Feasibility Trial

### Setting

Participants that fulfil trial inclusion criteria will be enrolled from participating secondary care centres. Three participating centres from different geographical regions have been chosen to enhance generalisability and applicability across the UK. Table 1 lists the hospital sites that will be set up to recruit patients into the randomised feasibility trial.

**Table 1:** PRESTO participating hospital trust sites

	Study sites
1.	Bart’s Health NHS Trust
2.	Leeds Teaching Hospitals NHS Trust
3.	Cardiff and Vale University Health Board

### Study participants

Patients are eligible to participate in this study if they:

1. Are aged 16 years or older;
2. Have a diagnosis of a high- or low-energy impact thoracolumbar vertebral body fracture, between T10 and L2, and confirmed by radiograph, computed tomography (CT) scan or magnetic resonance imaging (MRI) with at least ONE of the following criteria:
  - A kyphotic angle greater than 20° on standing radiographs, or if lying CT or radiograph then 15° of kyphosis; or
  - Reduction of vertebral body height by 25%; or
  - Fracture line propagating through the posterior wall of vertebra; or
  - Two contiguous vertebrae involved; or
  - Injury to the posterior longitudinal ligament (PLL) or annulus in addition to the body fracture

Patients will be excluded from this study if they:

1. Have an unstable fracture which obviously needs surgical stabilisation—decision made by the treating surgeon;
2. Have a spinal cord injury;
3. Have a pathological (other than osteoporotic) fracture, e.g. tumour/infection;
4. Are not considered suitable for surgery.

### Trial interventions

#### Non-surgical management (control) group

Non-surgical management in the control arm will consist of mobilisation in a brace or mobilisation without a brace, as recommended by the treating surgeon in consultation with the participant. The use and discontinuation of the brace is usually decided by the presence or absence of pain at the fracture site on mobilisation, but may also be for a predetermined prescribed period of time. The brace can be any orthotic device which supports the spine above and below the level of the fracture as considered appropriate by the treating surgeon, such as a thoracolumbar sacral orthosis (TLSO).

#### Surgical fixation (intervention) group

Currently in the UK, surgical treatment generally involves either open spinal surgery or minimally invasive surgery and both methods will be acceptable for use in the trial. Both procedures include placement of pedicle screws, but through different surgical approaches.

##### Open pedicle screw fixation

A midline approach is most commonly performed. Blood loss is minimised with diathermy dissection including careful haemostasis using bipolar and haemostatic agents. The junction between the pars interarticularis, lateral hemifacet and transverse process is identified and a starter awl, then pedicle finder is negotiated. A pedicle screw is placed within the pedicle, not too medial (potentially encroaching the spinal canal) or too lateral (potentially breaching the lateral wall and reducing the screw pullout strength). This confirmation is undertaken by direct palpation using a feeler and further confirmation can be achieved with intra-operative fluoroscopic imaging. Following screw placement, rods are locked into the screw heads stabilising the motion segment. Screws are placed in the un-fractured vertebrae either side of the fractured ones.

The surgeon may then decide to perform a spinal fusion by decorticating the bony surfaces and placing local bone graft and use bone substitutes as graft extenders. If surgical stabilisation alone is performed, decortication and graft placement is not undertaken.

##### Percutaneous pedicle screw fixation (minimally invasive surgery)

This follows the same principles. However, it is performed via multiple stab incisions on either side of the midline and is guided by intra-operative fluoroscopy. The advantages of percutaneous fixation are reduced blood loss and slightly reduced surgical time. Only surgical stabilisation can be undertaken with this technique and not spinal fusion.

Otherwise, the protocol follows that of the control group.

#### Rehabilitation

For both the surgical and non-surgical groups, patients will receive physiotherapy as per routine care. Details such as number of sessions and advice given by the physiotherapist will be recorded.

#### Imaging assessments

The routine imaging performed on admission will be used to confirm eligibility.

For the purposes of this trial, no imaging over and above usual care at a participating site will be requested. Information on the imaging undertaken for trial participants will be recorded.

### Primary outcome

The primary outcome will be recruitment rate, defined as the proportion of eligible participants who are randomised.

### Secondary outcomes

In order to investigate feasibility, we will collect data on the following:

(i) Recruitment

- Number of eligible patients;
- Proportion of eligible patients approached for consent;
- Proportion of eligible patients not approached for consent and reasons why;
- Proportion of patients approached who provide consent;
- Proportion of patients approached who do not provide consent and reasons why

(ii) Randomisation

- Proportion of patients providing consent who are randomised;
- Proportion of patients randomised who do not receive the randomly allocated treatment and reasons why

(iii) Cross-over

- Proportion of patients randomised to the non-surgical treatment who receive surgical management, at what time point and reasons why

(iv) Drop-out

- Proportion of patients dropping out between randomisation and follow-up at each time-point and reasons why

(v) Ability to collect clinical outcome measures

- Feasibility of gathering patient reported outcome measures and other outcome measures at baseline and follow-up (proportion of complete data for each outcome measure; proportion successfully gathered through the British Spine Registry)
- Feasibility of gathering data on complications and adverse events (proportion of complete data)

### Outcome measures relating to trial participants

Table 2 outlines the time points when the patient outcomes will be assessed. All participants will be followed up at 2 weeks and at 3 months post-randomisation. There will be an additional follow-up assessment 6 months post-randomisation for all patients recruited in the first 9 months of the recruitment period (approximately 2/3 of the total sample). The outcome measures are described below.

**Table 2:** Study assessment schedule

	STUDY PERIOD					
	Enrolment	Allocation	Post-allocation			
Time point	Baseline (pre-randomisation)	Randomisation	Intervention delivery	Week 2	Month 3	Month 6^1^
Enrolment:						
Eligibility screen	x					
Informed consent	x					
Demographic data	x					
Allocation		x				
Intervention:						
Non-operative management			x			
Surgical fixation			x			
Assessments:						
Oswestry Disability Index (ODI)	x (pre- and post-injury)				x	x
Visual analogue scale (VAS) for pain	x (post-injury)				x	x
Short Form-12 (SF12)	x (pre-injury)				x^2^	x^2^
EuroQol EQ-5D-5L	x (pre- and post-injury)				x	x
Patient & surgeon preferences	x					
Sagittal plane kyphosis	x			x	x	x
Treatment information				x	x	x
Basic health economics data (i.e. health care resource use)					x	x

^1^Only those patients that reach 6 months follow-up during period

^2^Via postal questionnaires only

#### Oswestry Disability Index

This is a commonly recommended patient-reported outcome measure for low back pain and spinal surgery [4–6] and is part of the outcome set used by the British Spine Registry. It assesses limitations across ten aspects of daily living (pain intensity, personal care, lifting, walking, sitting, standing, sleeping, sex life, social life and travelling) scored on a 0 to 5 scale [7]. Higher scores indicate higher levels of disability.

#### Visual analogue scale (VAS) for pain

The VAS for pain is a unidimensional measure of pain intensity which has been widely used in diverse adult populations [8, 9]. This is a continuous 11-point scale, anchored by two verbal descriptors with 0 representing ‘no pain’ and 10 representing ‘worst imaginable pain’, to measure average pain.

#### Short Form-12 (SF12)

This is a 12-item generic and widely used measure of physical and mental health completed by the participant, the population norms of which have a mean of 50 and standard deviation of 10; higher scores indicating better health [10]. The rationale for including the SF-12 is that it is feasible that a delay to return to work and recreational activities could impact on participants’ ability to perform other daily activities and their emotional well-being.

#### EuroQol 5 Dimensions–5 Levels Score (EQ-5D-5L)

The EQ-5D-5L is a validated generic patient-reported outcome measure [11]. The descriptive system has five health domains (mobility, self-care, usual activities, pain/discomfort, and anxiety/depression) with five response options for each domain (no problems, slight problems, moderate problems, severe problems and extreme problems). In addition, it has a health status visual analogue scale (VAS) which measures self-rated health with endpoints ranging from ‘the best health you can imagine’ to ‘the worst health you can imagine’.

Some patients taking part in the trial may not have capacity to complete baseline data. The EQ-5D has a separate proxy version. However, a recent systematic review suggested that proxy complexion within emergency and critical care settings does not generally give an accurate estimate of the patients EQ-5D [12, 13]. Therefore, in order to minimise bias in QALY estimation, patients that lack capacity will complete baseline EQ-5D respectively at earliest opportunity once capacity is gained.

#### Sagittal plane kyphosis

Kyphotic angle is measured using COBB technique. This involves measuring the angle between two lines parallel to the superior and the inferior end plate adjacent to the fractured vertebrae. This is measured using digital radiograph on the Picture Archiving and Communication System (PACS) or on computed tomographic (CT) images. If a CT is considered, the sagittal section through the mid-axial line should be used. Kyphotic angulation is considered to be a sign of instability.

#### Complications and adverse events

Information on all complications, additional surgery and adverse events will be collected in line with a study-specific standard operating procedure. Expected complications that will be recorded will include (but not be limited to) the following: death within 30 days of procedure, neurological complications, deep wound infection, superficial infection, rehospitalisation, implant failure, screw pull-out, re-operation and skin problems.

### Other outcomes

Length of hospital stay, return to work (time to return to work and whether individuals return to their previous job, a less physically demanding role, and whether there are any job modifications such as returning on reduced hours), and return to normal activities (e.g. volunteering, sports, hobbies).

### Data collection using the British Spine Registry

We will use the data collection tool from the British Spine Registry to collect patient-reported outcome data for patients that agree to receive questionnaires at 3 and 6 months via email to assess the viability (measured by proportion and completeness of data) of this method of data collection in any future definitive trial.

### Participant timeline

**Fig. 1:**
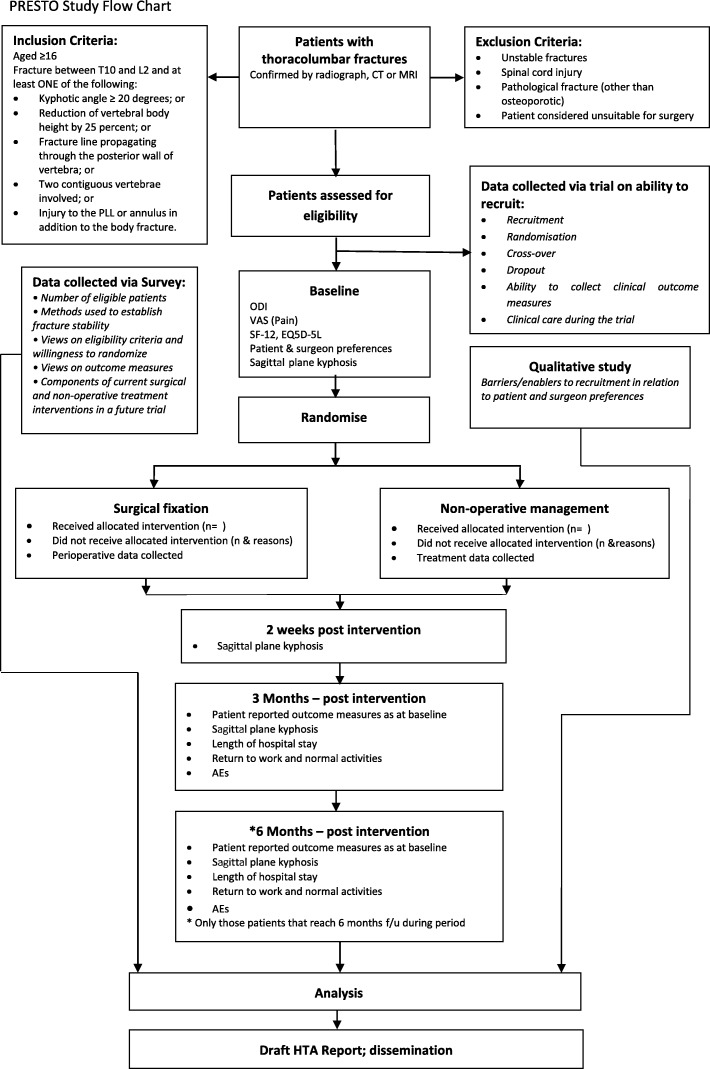
PRESTO study flow chart; figure illustrating the process of enrolling participants into the study, the interventions being compared, and timing of assessments for the participants in the trial

Figure 1 illustrates the process of enrolling participants into the study, the interventions being compared, and timing of assessments for the participants in the trial.

### Sample size

Based on initial discussions with the three recruiting centres, it is estimated that there will be at least 120 eligible patients during the 12-month recruitment period. A recruitment rate of 50% of eligible patients is considered appropriate for trials of this type which would give a sample size of 60. The identification of 120 eligible patients, will allow an estimation of a participation rate of 50% to within a 95% confidence interval of ± 9%. This is in line with the guidance for the sample size of feasibility/pilot studies which suggests there should be at least 12 participants in each arm of the study at the analysis stage to reliably estimate the standard deviation [14]. During the recruitment period, sites will identify and invite the maximum possible number of patients to participate, in order to generate the most robust estimate of recruitment rates possible.

### Recruitment

All patients diagnosed with a thoracolumbar vertebral body fracture between T10 and L2 will be assessed for eligibility. The research teams will work closely with the surgeons at each centre to optimise the screening and recruitment procedures for their local circumstances.

Patients will be provided with written information and given the opportunity to discuss and ask questions about the trial with research staff and their treating surgical team prior to making a decision on participation. Following confirmation of eligibility by the treating surgeon, written informed consent will be obtained by research staff prior to completion of baseline forms and randomisation. Participants will be free to withdraw from the study at any time without affecting their care.

Throughout the study, screening logs will be kept at each site to capture the number of patients assessed for eligibility and document reasons for exclusion. Patients who decline to participate or withdraw from the study will be given the opportunity to discuss/inform the research team of their reasoning behind their decision not to take part.

Strategies for achieving adequate participant enrolment to reach the target sample size include seeking advice from patient representatives, sharing best practice with site staff, feedback from qualitative interviews and regular discussion with Principal Investigators (PI).

Site staff will be provided with training at the site initiation visits to ensure adherence to the delivery of the interventions in the trial. During the trial, training and reminders will be implemented using email bulletins, discussion with the PIs and with Research Associates. In addition, the Trial Coordinators will provide support and guidance to staff when required (e.g. when new staff join or replace existing site staff) and will seek clinical guidance from the Chief Investigator when necessary.

### Consent

Written informed consent will be obtained, by an appropriately delegated member of the research team, from all patients considered able to make an informed decision about their participation in the research project.

A proportion of potential participants may be unconscious, distracted by their injuries, or may have had large doses of pain relief and therefore lack capacity to make an informed decision about participation. In these cases, an appropriate method, in line with the Mental Capacity Act (MCA) [15] and as approved by the Research Ethics Committee (REC), will then be used to gain prospective written agreement for the patient to be included in the trial from either a Personal or Nominated Consultee.

Where a Personal or Nominated Consultee has provided written agreement to participate on behalf of a participant, written consent to continue in the study will be sought from the participant at the first appropriate opportunity once they regain capacity. In the interim, best efforts will be made to involve participants who, temporarily or permanently lack capacity, to be involved in the decision about whether to take part in the study.

### Randomisation

Stratified block randomisation with stratification by centre and type of injury (high-energy trauma or low-energy osteoporotic) will be used to allocate participants on a 1:1 basis to surgery or non-surgical treatment.

When patients have given consent and their baseline forms have been completed, the Research Associate or recruiting surgeon will randomise them using the York Trials Unit’s (YTU) secure, web-based randomisation service, therefore ensuring allocation concealment and immediate unbiased allocation.

### Blinding

As the trial compares surgical versus non-surgical treatment, blinding of participants, surgeons and outcome assessors to treatment allocation is not possible. Participants will be informed of their allocations as will the clinical team managing each patient.

### Data management

Paper case report forms (CRFs) will be used to record all the information required from the protocol with the exception of Patient Reported Outcome Measures (PROMs) at 3 and 6 months. ODI, EQ-5D-5L, VAS and resource use for patients that agree to receive questionnaires via email will be obtained via the National Spine Registry platform and will be downloaded electronically at YTU.

All data will be completely anonymised for purposes of analysis and any subsequent reports or publications. For the purposes of ongoing data management, once randomised, individual participants will only be identified by trial identification numbers to maintain confidentiality.

All YTU data recorded electronically will be held in a secure environment at the University of York, with permissions for access in line with standard operating procedures.

All paper study documents held at YTU will be retained in a secure (kept locked when not in use) location for the duration of the trial.

All essential documents, including source documents, will be retained for a minimum period of 5 years after study completion.

Once YTU has completed the analysis and published all intended scientific journals, data will be made available for other researchers for secondary analysis upon request. Requests for access to data will be reviewed by the CI, study sponsor and trial team.

### Methods: survey of orthopaedic spine surgeons

A survey of NHS spine surgeons will be undertaken to estimate willingness to participate in a future trial, current caseloads of eligible patients, current practice in terms of establishing spinal stability and suitability for surgical or non-surgical management, methods of surgical fixation and non-surgical management currently used, and views on most important outcome domains for a full trial.

The electronic survey will be undertaken using Qualtrics, a secure web-based survey tool, which provides sophisticated software for use of question blocks, branch logic and flow through the survey. Best current evidence will be used to maximise response rate [16, 17]. The survey will be short, taking a maximum of 10 min to complete. Participants will be invited to participate through personalised emails, with a link to the survey. A formal invitation letter on letter-headed paper will also be attached. A reminder will be sent 2 weeks later, then another 1 week after that.

The questionnaire will be piloted in advance. It is anticipated that key aspects to be covered are likely to be as follows:

- Respondent and centre characteristics;
- Current management strategies for this population including methods to establish spinal stability and suitability for surgery or non-surgical treatment;
- Current methods of surgical fixation;
- Willingness to participate in a future trial and willingness to randomise based on the proposed inclusion criteria;
- Current caseload of eligible patients;
- Any centre factors that would need to be overcome to make recruitment to the trial possible

### Methods: qualitative study

A qualitative study will explore trial participants and surgeons’ views and experiences of the intervention and trial processes. Particular attention will be given to highlighting potential barriers and facilitators to recruitment and retention that could be used to inform the design of a full-scale trial. Semi-structured interviews will be conducted with patients who agree to take part in the trial, patients who decline participation, and surgeons and trial recruiters. Interviews will be undertaken face-to-face or by telephone according to which method is more convenient for individual participants. A flexible interview schedule will be developed following discussions with the research team, PPI team and surgeons with expertise in the area. All interviews will be audio-recorded with interviewee’s permission.

Up to 25 patients will be purposively selected from the cohort of patients who are eligible for recruitment into the feasibility trial. We aim to sample approximately eight to ten patients from each of the surgical and non-surgical treatment arms, in addition to approximately five patients who declined to participate in the feasibility trial to explore reasons for non-participation. Purposive selection will ensure maximum variation across the sample on the basis of age, gender trial site and treatment received.

Interviews will be conducted as soon after the invitation to participate in the study as practical to discuss in more detail the participants’ experiences of trial procedures, the intervention they were given and their recovery. We will specifically ascertain how the participants felt about the randomisation process being approached to take part in the trial. We will discuss the impact and acceptability of the intervention and clinical follow-up within the context of the daily lives of patients and their families to assess the interplay between the clinical intervention and their individual circumstances, for example, employment, housing and family composition.

In addition, 15 to 20 spinal surgeons from across the UK or staff recruiting patients to the feasibility study will be interviewed to discuss current practice regarding treatment of thoracolumbar fractures to determine facilitators and barriers to running a full-scale trial, and the potential of changing practice as a result of the findings. Initially, ‘key informants’ who have a particular expertise in thoracolumbar fractures will be recruited via the clinical collaborators on the project and from the feasibility trial sites. This will allow us to ascertain a broad overview of current practice in the field. Following the key informant interviews, snowballing techniques will be used to identify other clinicians. This approach is appropriate when the number of experts in the field is relatively small; therefore, many surgeons are known to each other. Further purposive recruitment will be through the identification of potential participants from the responses provided in the survey of orthopaedic spine surgeons to ensure that all views on the acceptability of treatment options, clinical equipoise and willingness to participate in a trial are captured. Surgeons will be asked about the feasibility/acceptability of providing either intervention on a regular basis, willingness to randomise, workload/staffing implications, training requirements, and readiness to employ the findings of a definitive trial into their normal practices.

### Statistical analysis of feasibility trial

A detailed analysis plan will be agreed with the Combined Trial Steering and Data Monitoring and Ethics Committee (Combined TSC/DMEC) at an early stage of the study, before all of the data has been collected. Any subsequent amendments will be clearly stated and justified.

A single analysis will be performed at the end of the trial using Stata v15 or later. Since this is a feasibility study, no formal statistical testing will be undertaken. Baseline data will be summarised by trial arm as randomised, with no formal comparisons between the groups. Continuous data will be reported descriptively (mean, standard deviation, median, minimum, maximum, and number missing), and categorical data by counts and percentages.

The recruitment rate will be reported monthly and overall, by hospital site and number of eligible patients will be summarised overall, by site, using counts and percentages.

The following will also be reported: the proportion of eligible patients approached for consent, proportion of eligible patients not approached for consent, proportion of eligible patients approached who consented, proportion of patients who did not provide consent, proportion of participants providing consent who were randomised, proportion of participants randomised who did not receive the randomly allocated treatment, proportion of participants who crossed over from non-surgical treatment to surgery and at what time point, and the proportion of participants dropping out between randomisation and follow-up. Explanations for these reported proportions will also be reported where available.

### Interim analysis

There are no planned interim analyses for the feasibility trial and no stopping guidelines.

### Economic evaluation

A full cost-effectiveness analysis will not be undertaken as part of this feasibility study. The economic evaluation will be used to identify data needed for an economic analysis as part of a full-scale trial. Individual participant data from the trial will be used to evaluate resource use, costs, and health outcomes associated with the interventions.

The acceptability of resource use questionnaires to capture the impact of care on the NHS and productivity will be assessed. The costing approach will be conducted from the National Health Services (NHS) and Personal Social Service (PSS) perspective. Health service resource use will be collected prospectively during the study using self-reported questionnaires and hospital forms at 3 and 6 months. Costs components will comprise all initial and subsequent inpatient episodes, outpatient hospital visits and A&E hospital admissions, and primary care visits (e.g. GP, nurse and physiotherapy). The total resource use will be calculated for each participant in both groups for the duration of the study. Health care resource use will be presented for both arms in terms of mean value, standard deviation, and mean difference (with 95% CI) between the groups. Unit costs will be derived from established national costing sources such as NHS reference costs [18], PSSRU unit costs of health and social care [19], and the British National Formulary [20].

The utility of participants will be measured using the EQ-5D-5L at baseline, 3 and 6 months. The raw EQ-5D -5L scores according to domain will be displayed, in order to examine the movements between levels for each domain according to the trial arm. Utility values will be estimated using the mapping function [21] according to the recent NICE statement on the use of the EQ-5D-5L [22].

The nature and amount of missing economic data will be explored which in turn will guide (i) on the sources to be used for the primary and secondary analyses in a full study, and (ii) on the imputation approach for the definitive trial.

### Analysis of survey of orthopaedic spine surgeons

Data will be downloaded from Qualtrics into Microsoft Excel. Standard checks will be undertaken to identify and remove errors, for example outliers, inconsistencies, and omissions. The response rate to the survey and individual questions will be calculated. Descriptive analyses of respondent characteristics will be undertaken to allow exploration of the representativeness of the sample. Descriptive analyses will be undertaken of responses to questions and summary statistics presented. A statistical analysis plan will be prepared before any data are downloaded and will outline any statistical comparisons that will be undertaken. Reporting will follow the CHERRIES guideline [23].

### Qualitative study analysis

The approach to analysis will be iterative and aided by the use of NVIVO (version 11) software. Initially, following transcription, interview material will be organised according to analytical headings using a constant comparison approach [24]. To introduce transparency and a systematic approach, we will engage in detailed familiarisation, identification and indexing of key themes, contextualising these themes in relation to the broader dataset, and interpreting them with a focus on addressing the specific aims of the study:

- Are surgeons willing to randomise eligible patients and adhere to randomisation to (i) surgical fixation or (ii) initial non-surgical management?
- Are patients willing to be randomised in a trial comparing the two treatments?
- Understand what methods of establishing spinal stability and clinical decision-making (suitability for surgery or non-surgical management) are currently used
- Understand what methods of surgical fixation and non-surgical management are currently being used
- What are the barriers to successful delivery of the future trial and how can they be overcome?

During the analysis, regular meetings will be held between the research team, and PPI participants where appropriate, to discuss the emergent themes from the fieldwork material. Findings from the qualitative work will be integrated with the feasibility trial outcomes and the survey in order to inform the design of a full-scale RCT.

### Overall synthesis of data

The data from the three elements of the study will be reported. The estimated recruitment rate and 95% confidence interval will be reported. Based on the data collated from the survey, the qualitative studies of patients and surgeons, and the additional data collected during the trial on any barriers to successful delivery of a future trial will be identified and recommendations made as to how they might be overcome. Recommendations will also be made regarding any changes required to the study design.

### Adverse event management

All adverse events (AE) requiring reporting (as defined by a study AE reporting procedure) will be reported on an AE form.

Non-serious, expected complications considered to be as a consequence of the surgery or conservative management, such as pain, and reported as complications on the designated forms will not be reported as adverse events in the context of this study.

Serious adverse events (SAEs) will only be reported if they appear to be related to an aspect of taking part in the study. Those events that are confirmed to be related to the research and are unexpected serious adverse events will be reported to the Research Ethics Committee (REC) within 15 days of the Chief Investigator becoming aware of the event.

The research nurse/physiotherapist will record all directly observed AEs and all AEs reported by the trial patient up to 3 months (and up to 6 months for those patients that reach this time point) following their trial treatment.

In addition, sites will be instructed to follow their own local procedures for the reporting of any adverse events linked to clinical care.

### Trial oversight

The day-to-day management of the trial will be the responsibility of the Trial Manager/Coordinator, based at York Trials Unit (YTU) and supported by other relevant members of unit staff. The trial statistician and health economist will be closely involved in setting up data capture systems and forms.

The Trial Management Group (TMG) is the executive decision making body and is responsible for overseeing the day-to-day running and management of the trial. The TMG will meet regularly, according to the needs of the study.

Due to the fact that this is a low-risk feasibility study with no planned interim analyses for either futility or safety, approval has been obtained from the funders to set up a Combined Trial Steering and Data Monitoring and Ethics Committee to undertake the roles traditionally undertaken separately by the TSC and the DMEC. The Combined TSC/DMEC will adopt a DAMOCLES charter which defines its terms of reference and operation in relation to oversight of the trial. The committee will meet at least annually or more frequently if the committee requests.

### Quality control

South Tees Hospitals NHS Foundation Trust has agreed to be the lead sponsor for this project and take overall responsibility for the quality of study conduct.

This study will be fully compliant with the Research Governance Framework (Health Research Authority, 2017b) and MRC Good Clinical Practice Guidance [25].

Review of key trial processes will be undertaken by the TMG which includes representation from the Sponsor. These meetings focus on aspects of patient recruitment (e.g. screening, consent, eligibility), allocation to study groups, adherence of the trial interventions to the protocol, monitoring of adverse events and reasons for patient withdrawal, and retention of trial participants. Independent review of the trial processes is undertaken by the Combined TSC/DMEC. The oversight committee advises on strategies to preserve the integrity of the trial where required.

## Discussion

Thoracolumbar fractures are an important public health problem but there is variation in practice regarding how they are treated and insufficient evidence from existing RCTs to support treatment decisions. The PRESTO trial is a rigorously designed study to assess the feasibility of conducting a full-scale trial which would contribute to the evidence base for informing clinical decisions for the treatment of this common fracture in adults.

### Protocol modifications

Any substantial amendments will be submitted to the HRA (and REC where required) having been agreed with the funding body, Sponsor, Combined TSC/DMEC and the TMG. Minor modifications to the protocol will be agreed with the TMG and Sponsor before submission for approval to HRA. All amendments will be implemented in the NHS organisations in agreement with the guidance and approval of the HRA. All amendments will be listed in the published final report to the funding body.

### Dissemination

This protocol is being made publically available.

Given that this is a feasibility study, our dissemination plan will reflect the fact that we will not be able to disseminate findings on the effectiveness of the intervention. Dissemination activities will focus on reporting the outcomes of the feasibility research to inform the future trial.

In addition to the detailed study monograph for the National Institute for Health Research Health Technology Assessment (HTA), the results will be disseminated in international, open-access peer-reviewed journals, through the local networks, and at national and international meetings in surgical care. The findings will also be disseminated to participants in the form of a plain English summary. Dissemination through patient websites such as the AfterTrauma website will be explored.

### Trial status

The current REC approved version of the protocol is version 1.1 (06 August 2018). This manuscript is a restructured and edited version of the current REC approved protocol to comply with the SPIRIT guideline. Recruitment into the PRESTO trial commenced in April 2018 and is ongoing at the time of manuscript submission. To date, 11 patients have been randomised (February 2019). Recruitment was originally scheduled to end in January 2019. An extension to the recruitment period was discussed and recommended by the TMG and TSC/DMEC and which was agreed upon by the funders. The recruitment phase has been extended to the end of March 2019.

The survey of surgeons was conducted between March 2018 and November 2018.

Recruitment into the qualitative study began in April 2018 and is ongoing at the time of manuscript submission. To date, three participant and 15 staff interviews have been conducted.

## Data Availability

Not applicable

## Abbreviations

CI: Confidence interval
CONSORT: Consolidated standards of reporting trials
CRF: Case report form
DMEC: Data Monitoring and Ethics Committee
EQ-5D: EuroQol 5 Dimensions
ISRCTN: International Standard Randomised Controlled Trial Number
MCA: Mental Capacity Act
NHS: National Health Service
ODI: Oswestry Disability Index
PROM: Patient-reported outcome measure
PSS: Personal social services
QALY: Quality-adjusted life year
RCT: Randomised controlled trial
REC: Research Ethics Committee
SF-12: Short Form 12-Item Questionnaire
TMG: Trial Management Group
TSC: Trial Steering Committee
VAS: Visual analogue scale
